# How adaptation currents and synaptic inhibition change threshold, gain and variability of neuronal spiking

**DOI:** 10.1186/1471-2202-14-S1-P299

**Published:** 2013-07-08

**Authors:** Josef Ladenbauer, Moritz Augustin, Klaus Obermayer

**Affiliations:** 1 Neural Information Processing Group, Berlin Institute of Technology, Berlin, Germany; 2 Bernstein Center for Computational Neuroscience Berlin, Berlin, Germany

## 

Many types of neurons show spike rate adaptation, a gradual decrease in spiking activity following a sudden increase in stimulus intensity. This behavior is typically mediated by slow potassium currents through voltage-sensitive low-threshold or calcium-activated high-threshold channels, both of which are susceptible to cholinergic modulation [1]. Such adaptation currents (and changes thereof) contribute to frequency selectivity [2], coding [3] and attention [4]. These effects are likely caused by altering the relationship between synaptic input and spike rate output (I-O curve) as well as the characteristics of inter-spike intervals (ISI). Here we investigate (i) how voltage-dependent subthreshold and spike-dependent adaptation currents change the neuronal I-O curve as well as the ISI distribution for different input statistics and (ii) how these changes compare to those induced by synaptic inhibition.

Based on a population of adaptive exponential integrate-and-fire (aEIF) model neurons receiving noisy external and recurrent synaptic inputs we use the Fokker-Planck equation to compute spike rates and ISI distributions in the limit of a large adaptation timescale.

We show that a subthreshold adaptation current or synaptic inhibition received from independent neurons (external inhibition) change the neuronal I-O curve subtractively. That is, both mechanisms increase the spike threshold. On the other hand, a spike-triggered adaptation current or inhibitory synaptic feedback (recurrent inhibition) change the I-O curve divisively, i.e., they reduce the neuronal gain, see Figure 1A. Both types of adaptation current naturally increase the mean ISI. Surprisingly, they affect spiking variability in opposite ways. Subthreshold adaptation leads to an increase while spike-triggered adaptation causes a decrease of variability, see Figure 1B. Both types of synaptic inhibition however increase spiking variability. We simplify the model by neglecting the leak conductance which allows to analytically derive expressions describing these effects. For validation purposes, we show that the effective description of subthreshold and spike-triggered adaptation in the aEIF model corresponds well to the biophysical description of a voltage-sensitive muscarinic and a calcium-activated potassium current in a Hodgkin-Huxley type neuron model, respectively. Our results suggest that neuronal adaptation currents differentially contribute to neuronal threshold and gain control as well as ISI-based coding schemes.

**Figure 1:**
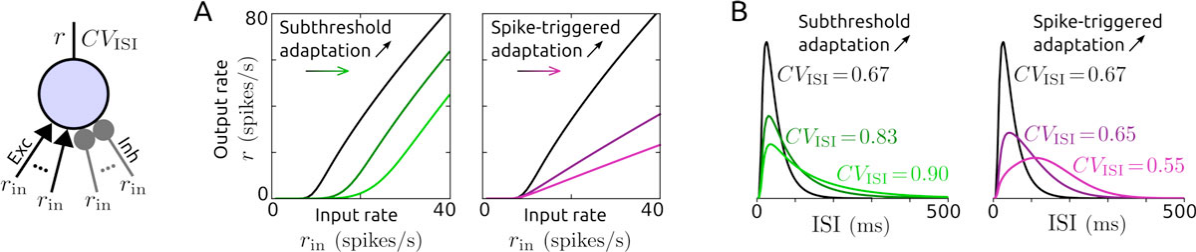
**Steady-state I-O relationships (**A**) and ISI distributions (**B**) for neurons without adaptation (black) and different degrees of subthreshold (green) or spike-triggered adaptation (magenta), driven by excitatory and inhibitory neurons whose spike times are generated by Poisson processes**.

## References

[B1] McCormick DA Neurotransmitter actions in the thalamus and cerebral cortex and their role in thalamocortical activity Progr Neurobiol 1992 39 337 388 10.1016/0301-0082(92)90012-4 1354387

[B2] Deemyad T Kroeger J Chacron M Sub and suprathreshold adaptation currents have opposite effects on frequency tuning J Physiol 2012 590 4839 4858 10.1113/jphysiol.2012.234401 22733663 PMC3487040

[B3] Prescott S Sejnowski T Spike-rate coding and spike-time coding are affected oppositely by different adaptation mechanisms J Neurosci 2008 28 50 13649 13661 10.1523/JNEUROSCI.1792-08.2008 19074038 PMC2819463

[B4] Herrero JL Roberts MJ Delicato LS Gieselmann MA Dayan P Thiele A Acetylcholine contributes through muscarinic receptors to attentional modulation in V1 Nature 2008 454 28 1110 1114 18633352 10.1038/nature07141 PMC2666819

